# Serum CCL18 May Reflect Multiorgan Involvement with Poor Outcome in Systemic Sclerosis

**DOI:** 10.3390/biom16010136

**Published:** 2026-01-13

**Authors:** Kristóf Filipánits, Gabriella Nagy, Dávid Kurszán Jász, Tünde Minier, Diána Simon, Szabina Erdő-Bonyár, Tímea Berki, Gábor Kumánovics

**Affiliations:** 1 Department of Rheumatology and Immunology, Medical School, University of Pécs, 7632 Pécs, Hungary; nagy.gabriella@pte.hu (G.N.); jasz.david@pte.hu (D.K.J.); minier.tunde@pte.hu (T.M.); kumanovics.gabor@pte.hu (G.K.); 2 Department of Immunology and Biotechnology, Medical School, University of Pécs, 7632 Pécs, Hungary; simon.diana@pte.hu (D.S.); erdo-bonyar.szabina@pte.hu (S.E.-B.); berki.timea@pte.hu (T.B.)

**Keywords:** systemic sclerosis, CCL18, serum biomarker, interstitial lung disease, multiorgan involvement, disease activity, prognosis, survival

## Abstract

Background: Serum C–C motif chemokine ligand 18 (seCCL18) in systemic sclerosis (SSc) has been primarily associated with progressive interstitial lung disease (SSc-ILD) and mortality. However, its relationship with non-pulmonary organ involvement, disease activity, and long-term outcome has not been comprehensively evaluated. We therefore examined the clinical relevance of seCCL18 in a single-center SSc cohort. Methods: A total of 151 patients with SSc (83 diffuse cutaneous (dcSSc), 68 limited cutaneous SSc (lcSSc); median (IQR) disease duration: 9 (4;16) years) and 47 age- and sex-matched healthy controls (HCs) were enrolled. Serum CCL18 concentrations were measured by enzyme-linked immunosorbent assay. Elevated seCCL18 was defined as >130 ng/mL (mean + 2 SD of the healthy control group). Organ involvement and disease activity (EUSTAR Activity Index, EUSTAR-AI) were assessed at baseline, while survival was analysed longitudinally. Results: Patients with SSc had significantly higher seCCL18 levels than HCs (mean ± SD: 99.9 ± 43.2 vs. 75.0 ± 27.5 ng/mL, *p* < 0.01). Elevated seCCL18 was associated with SSc-ILD (81.1% vs. 60.5%, *p* = 0.022), reduced forced vital capacity (FVC < 70%: 16.2% vs. 3.5%, *p* = 0.006), and reduced diffusing capacity for carbon monoxide (DLCO < 70%: 80.6% vs. 54.4%, *p* = 0.005). Higher seCCL18 levels were observed in patients with myocardial disease (104.8 ± 41.8 vs. 83.8 ± 44.2 ng/mL, *p* = 0.008), left ventricular diastolic dysfunction (107.1 ± 40.5 vs. 84.5 ± 45.0 ng/mL, *p* < 0.001), and oesophageal involvement (110.7 ± 38.3 vs. 93.3 ± 43.1 ng/mL, *p* = 0.009). SeCCL18 levels above the cut-off were more frequently associated with tendon friction rubs (51.4% vs. 27.4%, *p* = 0.007), active disease (EUSTAR-AI ≥ 2.5: 73% vs. 44%, *p* = 0.002), and elevated inflammatory markers (CRP > 5 mg/L: 51.4% vs. 19.3%, *p* < 0.001; ESR > 28 mm/h: 37.8% vs. 18.4%, *p* = 0.015). During a median follow-up of 87 months, 22 patients (15%) died. Elevated baseline seCCL18 predicted poorer survival in univariate analysis (log-rank *p* = 0.013) and remained an independent predictor of mortality in multivariable Cox regression (HR 1.789; 95% CI 1.133–2.824; *p* = 0.013), together with declining DLCO and reduced six-minute walk test performance. Conclusions: Elevated seCCL18 may identify patients with systemic sclerosis who exhibit a more severe multisystem phenotype, including cardiopulmonary, gastrointestinal, and musculoskeletal involvement, increased inflammatory activity, and reduced long-term survival. These findings suggest that seCCL18 may have some clinical utility as a prognostic biomarker reflecting widespread disease involvement beyond the lungs, even in patients with long-standing disease; however, the lack of an established cut-off value requires further validation in prospective, multicentre studies.

## 1. Introduction

Systemic sclerosis (SSc) is a systemic autoimmune disease, featuring autoimmunity, obliterative vasculopathy and tissue fibrosis [1]. In clinical practice, two main subtypes are distinguished: diffuse cutaneous systemic sclerosis (dcSSc) and limited cutaneous systemic sclerosis (lcSSc) [2]. The fourfold higher mortality risk compared to the general population, combined with its diverse clinical trajectories, places SSc among the connective tissue diseases with the most severe prognosis and impaired quality of life [3,4,5]. Despite recent advances in the understanding of disease pathogenesis, reliable biomarkers for routine disease monitoring and clinical management remain limited.

Investigation of serum biomarkers has become essential for exploring the pathomechanisms of fibrosis. Krebs von den Lungen-6 (KL-6), surfactant protein-D (SP-D) and interleukin 8 (IL-8) are associated with systemic sclerosis-associated interstitial lung disease (SSc-ILD), while matrix metalloproteinases 9 and 12 (MMP-9, MMP-12), lysyl oxidase (LOX), are linked to skin fibrosis, and chemokine (C-X-C motif) ligand 4 is connected to both [6,7,8,9,10,11,12].

In our paper, we focus on seCCL18, a promising biomarker consistently associated with progressive SSc-ILD, a major determinant of mortality. Although other severe organ manifestations are independent risk factors for higher mortality as well, there is currently no convincing evidence linking seCCL18 to these non-pulmonary clinical features [13,14,15,16,17,18]. Among several candidate serum biomarkers, the seCCL18 is of particular interest because it is predominantly secreted by alternatively activated macrophages in fibrotic tissues and reflects macrophage-driven inflammation and extracellular matrix turnover [19,20,21,22,23]. Beyond SSc, seCCL18 has also been linked to a variety of other systemic autoimmune and connective tissue diseases. In rheumatoid arthritis, increased CCL18 expression in both serum and synovial tissue has been associated with disease activity [24,25]. Elevated seCCL18 levels have been observed in IgG4-related disease, where they were associated with disease activity and reflect the underlying fibro-inflammatory process [26]. The seCCL18 is also highly expressed in patients with antineutrophil cytoplasmic antibody (ANCA)–associated crescentic glomerulonephritis, where it reflects crescent formation, interstitial inflammation, and impaired renal function [27]. Beyond autoimmune diseases, seCCL18 was a strong predictor of progression and mortality in idiopathic pulmonary fibrosis [28]. In SSc, concurrently with other studies, in a large European cohort, the seCCL18 appeared to be a predictive biomarker for the progression of ILD (based on the FVC decline), and the high baseline concentration was found to be associated with progressive SSc-ILD and reduced survival [29,30,31]. According to a large German single-centre cohort, seCCL18 performed well as a diagnostic biomarker for SSc-ILD [32]. In an Australian cohort with 407 SSc patients, seCCL18 was found to be associated with increased mortality [33]. Patients treated with mycophenolate-mofetil or cyclophosphamide showed a significant decline in the seCCL18 levels after one year of treatment [34]. The seCCL18 levels were lower in patients treated with tocilizumab compared to placebo in the faSScinate Phase 2 randomised clinical trial investigating the effectiveness of subcutaneous tocilizumab [35].

However, data on its role beyond pulmonary involvement and its independent prognostic value remain limited. This study aimed to comprehensively describe the associations between elevated seCCL18 levels and mortality as well as the concurrent presence of different organ involvements in SSc.

## 2. Materials and Methods

We conducted a cross-sectional study with longitudinal survival analysis in a well-characterized single European Scleroderma Trials and Research Group (EUSTAR) center SSc cohort. This study was designed in accordance with the Strengthening the Reporting of Observational Studies in Epidemiology (STROBE) recommendations (Supplementary Table S1) [36].

### 2.1. Ethics

Written informed consent was obtained from all subjects prior to the study, in accordance with the Declaration of Helsinki. Ethical approval for this study was obtained from the Hungarian National Ethics Committee (Approval No. 30636-3/2017/EKU, Approval Date: 27 June 2017).

### 2.2. Patients and Controls

One hundred and fifty-one Caucasian SSc patients, predominantly female (129 female, 22 male), were prospectively enrolled at our single tertiary-care EUSTAR centre (Department of Rheumatology and Immunology, University of Pécs Medical School, Pécs, Hungary) between 2017 and 2020. All patients fulfilled the American College of Rheumatology (ACR)/European League Against Rheumatism (EULAR) Criteria (2013) for SSc [37]. Patients were categorised into dcSSc and lcSSc subtypes [2,38]. More than half of the patients (*n* = 83, 55%) belonged to the dcSSc group. Disease duration in years was estimated based on the elapsed time between the onset of the first non-Raynaud symptom of SSc and the date of the investigation. The median (IQR) disease duration of the cohort was 9 (4;16) years. Thirty-two patients (21.2%) had a disease duration of ≤3 years, including 23 patients with dcSSc. SSc patients with coexistent systemic autoimmune syndrome were also included in this study. Detailed physical examination involving the skin, vascular, musculoskeletal, pulmonary and cardiac systems was performed based on an established protocol according to the EUSTAR standards. As a control group, 47 age- and sex-matched healthy individuals (HCs) were enrolled.

### 2.3. Organ Involvement

Organ systems were screened for manifestations (respiratory, cardiac, renal, musculoskeletal, skin, vascular, and gastrointestinal) in accordance with current clinical practice recommendations. SSc-ILD was defined by high-resolution computed tomography (HRCT) scans interpreted by two trained radiologists [39]. Contractures of the small and large joints were defined if their range of motion was <75% upon physical examination, evaluated by experienced physical therapists (Bálint, Z., et al. [40]). Sicca symptoms (dry eyes or dry mouth) were diagnosed based on the first two items of the American European Consensus Group (AECG) criteria for Sjögren’s Syndrome [41]. Upper GI involvement was diagnosed in patients who presented with at least one of the following: oesophageal stricture or dysmotility on oesophageal barium swallow test, gastroesophageal reflux disease (GERD), or gastric vascular ectasia, as evaluated by upper GI endoscopy [42]. Intestinal pseudo-obstruction and significant weight loss and/or low BMI due to malabsorption were considered lower GI complications of SSc, diagnosed by abdominal X-ray or CT, colonoscopy, hydrogen breath test or simply by history taking [42]. Pulmonary arterial hypertension (PAH) was diagnosed if mean pulmonary arterial pressure (mPAP) ≥ 25 mmHg, pulmonary artery wedge pressure (PAWP) ≤ 15 mmHg, and pulmonary vascular resistance (PVR) > 3 Wood units (WU) were verified by right heart catheterisation (RHC) in the absence of significant interstitial lung disease [43]. Myocardial disease attributable to SSc was defined based on clinical features and investigations, in which arrhythmias, conduction abnormalities (bundle branch block or atrioventricular block) on 12-lead electrocardiography (ECG) or 24 h Holter monitoring, and systolic or diastolic dysfunction on transthoracic echocardiography (TTE) were evaluated by an experienced cardiologist [44]. The presence of pericardial effusion was assessed by TTE as well [44]. Scleroderma renal crisis was defined according to currently available definitions and clinical symptoms [45]. Arthritis was defined as the presence of tenderness and swelling in at least one joint on physical examination, attributable to SSc, after excluding alternative causes. The 6 min walk test (6MWT) was performed according to standard protocol [38], and the distance covered with oxygen saturation data was recorded along with standardised reference values [46].

### 2.4. Biomarker Tests

Blood samples were collected at the time of enrolment. The seCCL18 levels were measured by commercially available enzyme-linked immunosorbent assay (ELISA) kits (Human CCL18/PARC Immunoassay Cat. No.: DCL180B, R&D Systems Inc., Minneapolis, MN, USA) according to the manufacturer’s protocol. Abnormally elevated seCCL18 levels were defined as values exceeding the mean + 2 standard deviations (SD) of the healthy controls. Autoantibodies were assessed using the Quanta Lite ANA ELISA kit (Inova Diagnostics, San Diego, CA, USA). Anti-topoisomerase I (ATA) antibodies were measured with the ORG 514 ELISA assay (Orgentec, Mainz, Germany), and anti-centromere antibodies (ACA) were determined using the ORG 633 ELISA assay (Orgentec, Mainz, Germany). RNA-Polymerase III (RNA-Pol III) autoantibodies were analysed with the Euroimmun DL 15,321,601 immunoblot assay (Euroimmun, Mountain Lake, VA, USA).

### 2.5. Survival Analysis

Survival data were retrieved from the Hungarian National Health Insurance Fund (NHIF) database and verified using the institutional electronic medical record (EMR) system. Follow-up time was calculated from the date of enrolment and serum sampling to either the date of death or the last available record in the database; the final database check was performed on 30 June 2025.

### 2.6. Disease Activity

Disease activity was assessed using the revised European Scleroderma Trials and Research Group (EUSTAR) Activity Index (EUSTAR-AI) [47].

### 2.7. Statistical Analysis

Statistical analyses were performed using SPSS software (version 30.0, IBM Corp., Chicago, IL, USA). Normality of continuous variables was assessed using exploratory normality testing. For descriptive purposes, data are presented as mean ± standard deviation or median (interquartile range),depending on the distribution of the given parameter. Due to heterogeneous distributions and small subgroup sizes, non-parametric statistical tests were applied consistently for all group comparisons, irrespective of the results of normality testing. The chi-square (χ^2^) test and Fisher’s exact test were applied to compare categorical variables, as appropriate. The Mann–Whitney U test and Kruskal–Wallis test with post hoc Dunn–Bonferroni correction were used for comparisons of continuous variables. Spearman’s rank correlation was conducted to assess relationships between continuous variables. Only correlation coefficients ≥ 0.4 with *p* < 0.05 were considered clinically relevant. Survival analyses were performed using Kaplan–Meier estimates and Cox proportional hazards regression models with 95% confidence intervals (CI). Figures were generated using GraphPad Prism (version 9.0, GraphPad Software, Inc., San Diego, CA, USA).

## 3. Results

### 3.1. Baseline Characteristics and Organ Manifestations

Detailed clinical characteristics of the total SSc cohort, including subtype analysis, are shown in Table S2.

Female patients were more prevalent in the lcSSc subgroup than in the dcSSc subgroup (92.6% vs. 79.5%, *p* = 0.023). Patients with lcSSc were older at enrolment (median (IQR) 61 (53.25;65) vs. 54 (40;63) years, *p* = 0.002) and had a longer disease duration (median (IQR) 12.5 (6.25;17) vs. 7 (3;13) years, *p* = 0.001). Distinct serological patterns were observed between subtypes. ACA antibodies were characteristic of lcSSc (36.8% vs. 12.0%, *p* < 0.001), whereas ATA positivity was more frequent in dcSSc (38.6% vs. 11.8%, *p* < 0.001). Immunosuppressive therapy was used more commonly in dcSSc compared with lcSSc (62.7% vs. 30.9%, *p* < 0.001).

SSc-ILD was more prevalent among dcSSc patients than in lcSSc (72.3% vs. 57.4%, *p* = 0.008). In contrast, arterial hypertension (61.8% vs. 37.3%, *p* = 0.003), myocardial disease (85.3% vs. 69.9%, *p* = 0.026) and left ventricular diastolic dysfunction (77.9% vs. 60.2%, *p* = 0.020) were more common in lcSSc. Vascular and musculoskeletal manifestations also showed differences: digital ulcers (DUs) (45.8% vs. 26.5%, *p* = 0.015) and tendon friction rubs (42.7% vs. 22.1%, *p* = 0.008) occurred more frequently in dcSSc. Skin involvement differed markedly between subtypes. The median (IQR) mRSS was significantly higher in dcSSc (*p* < 0.001), and a more extensive skin thickening (mRSS > 14) was present only in dcSSc (31.3% vs. 0%, *p* < 0.001).

The HCs (*n* = 47) were predominantly female (81%), with a median (IQR) age of 55 (41;61) years.

### 3.2. Demographics and Disease Duration

**Figure 1:**
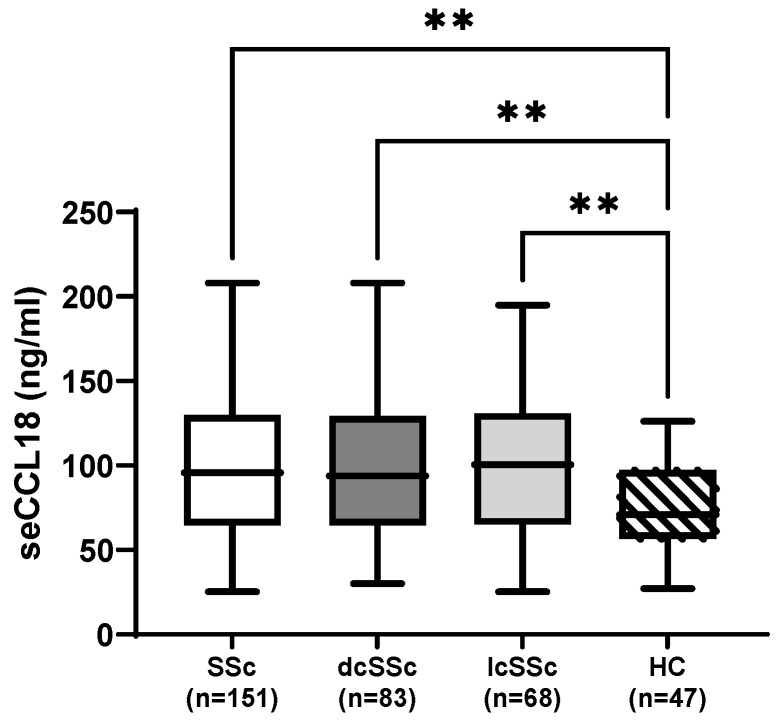
Serum CCL18 levels in patients with systemic sclerosis and healthy controls. seCCL18: serum CC chemokine ligand 18; SSc: systemic sclerosis; dcSSc: diffuse cutaneous systemic sclerosis; lcSSc: limited cutaneous systemic sclerosis; HC: healthy controls. Data are shown as box-and-whisker plots indicating the median, interquartile range, and minimum–maximum values. *p*-values were determined by the Kruskal–Wallis test. Asterisks represent statistical significance levels: *p* < 0.01 (**).

Elevated serum CCL18 was defined as values exceeding the mean + 2 SD of healthy controls (>130 ng/mL). The seCCL18 levels were higher in all SSc groups (total cohort, dcSSc, lcSSc) than in HCs (all *p* < 0.01; Figure 1). However, no significant difference was observed between dcSSc and lcSSc (Figure 1). Elevated seCCL18 levels were found in 37 (24.5%) SSc subjects. In the total cohort, no clinically relevant correlation was observed between seCCL18 levels and age (rho = 0.326, *p* < 0.01). Female SSc patients (*n* = 129) had significantly higher seCCL18 concentrations than males (*n* = 22) (mean ± SD: 102.66 ± 49.3 vs. 84.0 ± 49.3 ng/mL, *p* = 0.037). Female patients were also significantly older than males at enrolment (median (IQR) 60 (49;65) vs. 48.5 (30.75;59.25) years, *p* = 0.002) and at diagnosis (median 48 (39;54) vs. 41 (28;49.25) years, *p* = 0.021), whereas disease duration did not differ significantly between females and males (median (IQR) 10 (4;16.5) vs. 6 (2.75;10.75), *p* = 0.063).

SeCCL18 levels were higher in SSc than in healthy controls (75.0 ± 27.5 ng/mL), both in early (≤3 years: 101.9 ± 53.7 ng/mL, *p* = 0.047) and long-standing disease (>3 years: 99.4 ± 40.1 ng/mL, *p* < 0.001), with no difference between disease-duration subgroups and no correlation with disease duration. Among early patients, higher seCCL18 was observed in dcSSc (115.2 ± 55.7 vs. 75.0 ± 27.5 ng/mL, *p* = 0.016), but not in lcSSc.

### 3.3. SSc-ILD and Functional Exercise Capacity

Patient with elevated seCCL18 showed a higher rate of interstitial lung disease (ILD) compared to those with normal seCCL18 levels (81.1% vs. 60.5%, *p* = 0.022). Similarly, reduced FVC (<70%) and DLCO (<70%) were more common in the elevated seCCL18 group (16.7% vs. 3.5%, *p* = 0.006 and 80.6% vs. 54.4%, *p* = 0.005, respectively). In the dcSSc subset, elevated seCCL18 levels were also linked to SSc-ILD (94.7% vs. 65.6%, *p* = 0.013) and reduced DLCO (<70%) (84.2% vs. 57.8%, *p* = 0.035). In lcSSc, these patterns were observed but did not reach statistical significance (Table 1).

In patients with elevated seCCL18 levels, SSc-ILD was more frequently observed in the early SSc group than in those with longer disease duration (*n* = 9/9, 100% vs. *n* = 18/28, 64%, *p* = 0.036).

Patients with elevated seCCL18 had lower lung function values in the total cohort, including reduced FVC% (*p* = 0.006), DLCO% (*p* = 0.015) and TLC% (*p* = 0.002). In dcSSc, patients with elevated seCCL18 had significantly lower TLC% than those with normal seCCL18 (*p* = 0.004). In the lcSSc group, those with elevated seCCL18 had significantly lower FVC% (*p* = 0.007) (Table S3).

Patients with elevated seCCL18 levels and SSc-ILD (*n* = 30) had significantly lower FVC% (mean ± SD 91.0 ± 19.1 vs. 102.3 ± 22.4; *p* = 0.019) and TLC% (mean ± SD 100.3 ± 17.6 vs. 111.6 ± 21.1; *p* = 0.012) compared with patients with normal seCCL18 levels and SSc-ILD (*n* = 69), while DLCO% was lower but did not reach statistical significance.

Detailed seCCL18 concentrations according to respiratory involvement, including subset analyses, are shown in Figure S1a,b and provided in Supplementary Table S4.

Patients with FVC below 70% and DLCO below 80% had significantly higher seCCL18 levels (*p* = 0.023 and *p* = 0.004, respectively). In the dcSSc subset, both SSc-ILD and lower DLCO were linked to higher seCCL18 levels (*p* < 0.001 and *p* = 0.002, respectively) (Table S4).

The associations between functional exercise capacity, as measured by the 6MWT, and seCCL18 are presented in Table S3.

### 3.4. Non-Pulmonary Organ Involvement

Detailed seCCL18 concentrations according to organ involvement, autoantibody profile, and inflammatory parameters are provided in Supplementary Table S4.

#### 3.4.1. Cardiac

Higher seCCL18 levels were associated with arterial hypertension (*p* = 0.042), myocardial disease (*p* = 0.008), and left ventricular diastolic dysfunction (*p* < 0.001) in SSc, with the latter two associations remaining significant in the dcSSc subgroup (*p* = 0.011 and *p* = 0.003, respectively) (Table S4).

#### 3.4.2. Gastrointestinal

Patients with oesophageal involvement (dysmotility and/or stricture) had higher seCCL18 levels (*p* = 0.009). In dcSSc, the presence of GERD was associated with lower seCCL18 levels (*p* = 0.034) (Table S4).

Interestingly, GERD was more common in patients with normal seCCL18 levels in the total SSc cohort (79.8% vs. 59.5%; *p* = 0.013) (Table 2).

#### 3.4.3. Musculoskeletal and Skin

Musculoskeletal symptoms were more common among patients with elevated seCCL18, including small joint contractures (*p* = 0.029) and TFRs (*p* = 0.007). This association remained in both dcSSc (*p* = 0.040) and lcSSc (*p* = 0.045) subsets. In lcSSc, elevated seCCL18 levels were associated with DUs (*p* = 0.049) (Table 2).

#### 3.4.4. Laboratory Parameters

Laboratory markers of inflammation were also higher in patients with elevated seCCL18 (>130 ng/mL) levels, as both CRP > 5 mg/L (51.4% vs. 19.3%; *p* < 0.001) and ESR > 28 mm/h (37.8% vs. 18.4%; *p* = 0.015) were more frequently observed in this group (Table 2). Additionally, the presence of ATA was more frequent in the total cohort in patients with elevated seCCL18 levels (40.5% vs. 21.9%; *p* = 0.026). In lcSSc, elevated seCCL18 levels were associated with ATA positivity (27.8% vs. 6.0%; *p* = 0.014), and RNA polymerase III antibody positivity (22.2% vs. 4.1%; *p* = 0.040) (Table 2).

The seCCL18 levels showed a moderate positive correlation with both CRP (rho = 0.441, *p* < 0.001) and ESR (rho = 0.422, *p* < 0.001).

ATA positive patients had significantly higher seCCL18 levels (*p* = 0.015), particularly in the lcSSc subset (*p* = 0.019). In contrast, ACA positivity was associated with lower seCCL18 levels in dcSSc (*p* = 0.037). A small proportion of lcSSc patients, who were RNA-Pol-III positive had markedly higher seCCL18 levels compared to RNA-Pol-III negative lcSSc subjects (*p* = 0.004). Patients with CRP > 5 mg/L had higher seCCL18 levels in the total SSc cohort (*p* < 0.001) and across both subsets (*p* < 0.001). Patients with ESR > 28 mm/h also had higher seCCL18 levels in the total cohort (*p* < 0.001) and in both subsets (*p* = 0.013) (Table S4).

#### 3.4.5. Other

Sicca symptoms were more frequent among patients with normal seCCL18 concentrations in the total cohort and particularly in the lcSSc subset (66.7% vs. 45.9%, *p* = 0.025) (Table 2).

Patients with elevated seCCL18 were also more likely to be receiving immunosuppressive therapy at baseline (67.6% vs. 42.1%, *p* = 0.007) (Table 2).

Neither categorical stratification based on elevated seCCL18 levels nor analyses using absolute seCCL18 concentrations revealed associations with other assessed vascular, cardiac, cutaneous, musculoskeletal, or gastrointestinal manifestations, either in the total cohort or within the main SSc subsets.

### 3.5. Disease Activity

According to the EUSTAR-AI, half of the patients (51%) had an active disease at the time of enrolment (EUSTAR-AI ≥ 2.5). In the dcSSc subset 64% of the patients, in the lcSSc subset 35% were active (EUSTAR-AI ≥ 2.5).

Patients with elevated seCCL18 levels had an active disease more frequently based on the EUSTAR-AI (*n* = 27/37, 73%, vs. *n* = 50/114, 44%, *p* = 0.002). When analysed separately by SSc subtypes, a similar pattern was observed. In the dcSSc group, elevated seCCL18 levels were associated with a higher frequency of active disease according to EUSTAR-AI (*n* = 17/19, 89% vs. *n* = 36/64, 56%; *p* = 0.008). Likewise, in the lcSSc group, patients with elevated seCCL18 levels more frequently had active disease (*n* = 10/18, 55.5% vs. *n* = 14/50, 28%; *p* = 0.036).

In the total cohort, patients with active disease had higher seCCL18 levels (*n* = 77, 109.2 ± 46.5 ng/mL vs. *n* = 74, 90.3 ± 37.2, *p* = 0.009). This difference was only observed in the dcSSc subset (*n* = 53/83, 109.4 ± 48.7 vs. *n* = 30/83, 81.7 ± 32.1, *p* = 0.010) but not among patients with lcSSc.

### 3.6. Survival Analysis

The study cohort consisted of 151 patients, of whom 147 were eligible for survival analysis. Four patients were excluded due to insufficient follow-up information. During the follow-up (median (IQR) follow-up time: 87 (84;92) months) 22/147 patients (15%) died. Elevated baseline seCCL18 predicted poorer survival based on univariate analysis (Kaplan–Meier) (Log Rank = 6.218, *p* = 0.013) along with other known poor prognostic factors (Table 3). In our cohort, univariate Kaplan–Meier analysis showed no association between survival and the presence of myocardial disease, moderate or severe GI involvement (UCLA-GIT 2.0 score > 0.5), positivity for any SSc-specific autoantibodies (ATA, ACA or RNA-Pol III), extensive skin involvement (mRSS > 14), male sex, dcSSc subtype, reduced FVC (FVC < 70%), PAH, oesophageal involvement, or elevated inflammatory markers (CRP > 5 mg/L or ESR > 28 mm/h).

In the multivariate Cox regression, the independent predictors of mortality included: seCCL18 per 1-standard deviation increase (HR 1.789, 95% CI 1.133–2.824, *p* = 0.013), decreasing DLCO for each 10% drop, and reduced performance in the 6MWT for each 10% decline (all *p* < 0.05) (Table 3).

Variables removed: gender (*p* = 0.709), age at diagnosis (*p* = 0.482), BMI (*p* = 0.212), dcSSc subtype (*p* = 0.649), PAH (*p* = 0.104), CRP (*p* = 0.912), ESR (*p* = 0.384), presence of small joint contractures (*p* = 0.863), mRSS per 10 points increment (*p* = 0.158). The seCCL18 levels were standardised, and the results represent one standard deviation increase in the seCCL18 levels. Results of the multivariate analysis are presented as hazard ratios (HR) with 95% confidence intervals (CI). Statistically significant *p*-values (*p* < 0.05) are indicated in bold.

## 4. Discussion

In this cross-sectional study with survival analysis, we evaluated the clinical relevance of seCCL18 levels in a single EUSTAR-centre cohort of patients with SSc. Our findings confirm and extend prior evidence that seCCL18 is a significant biomarker of key disease processes in SSc, particularly macrophage-driven inflammation and tissue scarring. In SSc, increased seCCL18 may reflect a dual immunological role, with predominance of Th2 cells, M2 macrophages, and regulatory T cells promoting fibrotic processes, while reducing Th1-, Th17-, and M1-mediated inflammatory pathways, highlighting its ambiguous role [48]. We found elevated seCCL18 levels in nearly a quarter of the cohort, despite the relatively long median disease duration of 9 years. These levels were consistently linked to more severe organ involvement, decreased pulmonary function, and unfavourable outcomes. Our findings are in line with previous European and Australian cohort studies, further supporting seCCL18 as a clinically meaningful biomarker in SSc [32,33,49].

Consistent with earlier studies, patients with SSc had higher seCCL18 levels compared to HCs in our study. However, the literature reports a wide range of seCCL18 cut-off values, including both lower and higher thresholds than those applied in our cohort [30,49]. Notably, the increase in seCCL18 was independent of the SSc subset or its duration, suggesting that seCCL18 reflects underlying disease mechanisms rather than overall organ damage. The strong link between elevated seCCL18 and SSc-ILD supports its utility as a biomarker of pulmonary fibrosis. Patients with higher seCCL18 levels exhibited worse pulmonary function test results, including lower FVC(%), DLCO(%), and TLC(%) values. These findings support prior longitudinal studies demonstrating that elevated baseline seCCL18 predicts SSc-ILD progression and poor long-term pulmonary outcomes [21,30,34,49]. Notably, lcSSc-ILD patients had higher seCCL18 levels than dcSSc patients without ILD in our cohort, which supports that elevated seCCL18 is not limited to the dcSSc subtype. This may indicate that seCCL18 levels rise in the presence of SSc-ILD also in lcSSc. These findings indicate that elevated seCCL18 levels may serve as a marker of the presence of SSc-ILD in both SSc subsets, including patients with long-standing disease. In addition, higher seCCL18 levels were associated with myocardial involvement and hypertension, which was more common in the lcSSc group. Given that previous studies have also linked CCL18 to arterial hypertension [50], the higher prevalence of hypertension in lcSSc may partially explain the elevated seCCL18 levels in this subgroup, although this requires further investigation, particularly given that the prevalence of arterial hypertension increases with ageing [51].

While earlier studies have focused mainly on seCCL18 as a biomarker associated with ILD, lung function decline, and mortality, our results indicate that elevated seCCL18 also relates to more extensive disease severity [21,29,30,31,33,34,49,52]. Higher seCCL18 levels were associated with cardiac involvement—particularly myocardial disease and diastolic dysfunction—as well as specific gastrointestinal manifestations, such as oesophageal dysmotility, suggesting that seCCL18 reflects a broader fibrotic and inflammatory burden. In line with this, several previously confirmed manifestations associated with poor prognosis also showed significant associations with high baseline seCCL18 levels in our cohort [13,18,53,54]. The association with multiple EUSTAR-AI components further supports that seCCL18 may capture ongoing disease activity even in patients with long-standing SSc. Taken together, these findings indicate that seCCL18 could help identify clinically active patients beyond the early disease phase, including those who may require closer monitoring or active immunosuppressive treatment.

Interestingly, in patients with normal seCCL18 levels, GERD was more frequent despite widespread use of proton pump inhibitors or H2-receptor blockers, indicating clinically more severe and treatment-refractory reflux, and sicca symptoms were also more common in this group. The higher prevalence of these manifestations along with normal CCL18 levels may relate to mechanisms previously described, including an autopsy study showing predominant smooth muscle atrophy rather than fibrosis, as well as studies implicating M3 muscarinic receptor-blocking antibodies, neuropathy, and myopathy in the pathogenesis of GI manifestations in SSc [55,56,57,58]. Beyond this, our work is the first to systematically analyse seCCL18 in relation to the EUSTAR-AI and to a broad spectrum of non-pulmonary organ manifestations.

Survival is a hard endpoint in SSc, and there remains an unmet need to identify reliable prognostic biomarkers. In our survival analysis, elevated baseline seCCL18 levels emerged as an independent risk factor for mortality. Together with its association with multiorgan involvement, this indicates that seCCL18 may help identify patients with a broader, clinically meaningful disease burden. However, as only a small proportion of patients died during the 7-year follow-up and several well-known prognostic factors did not emerge as significant predictors of survival, our findings likely reflect both the substantially longer disease duration of our cohort and the shift toward earlier, more intensive management of high-risk individuals, factors that may attenuate the impact of traditional prognostic markers. Therefore, further investigations on a larger scale are needed to determine its prognostic role for overall mortality.

This study has several notable strengths. Firstly, a relatively high proportion of enrolled patients belong to the dcSSc subtype, which carries a worse prognosis and potentially more extensive internal organ involvement. Secondly, an additional strength of our study is the integration of survival data with minimal loss to follow-up, allowing for a reliable assessment of long-term outcomes. Furthermore, the association between elevated seCCL18 levels and mortality was confirmed not only in univariate analysis but also as an independent prognostic factor in multivariate Cox regression. This demonstrates that seCCL18 provides prognostic information beyond established functional and clinical predictors. Lastly, the comprehensive organ involvement assessment was characterised by minimal missing data, which strengthens the reliability of this study.

However, we are aware of some limitations. First, in this study, the relatively small sample sizes in some subgroup analyses represent a limitation commonly encountered in single-centre research. Second, the cross-sectional design prevents evaluation of longitudinal treatment effects on seCCL18, lung function changes, and ILD progression, even though prior data indicate that immunosuppression may lower seCCL18 levels [35]; however, such analyses are planned with follow-up data in future work. In addition, large, multicentre studies are required to establish clinically meaningful cut-off values and to validate the utility of seCCL18 at the individual patient level. Prospective studies with longitudinal follow-up will also be essential to clarify whether longitudinal changes in seCCL18 reflect disease activity over the natural course of the disease, and to what extent these changes may be influenced by therapeutic interventions. Finally, it should be emphasised that serum PARC/CCL-18 assays are not yet standardised; therefore, absolute concentrations may not be directly comparable across studies.

From a clinical perspective, seCCL18 assessment may provide complementary information on disease burden and prognosis in systemic sclerosis, particularly for identifying patients who may benefit from closer cardiopulmonary monitoring, while acknowledging that its routine use requires further validation.

## 5. Conclusions

In summary, our study demonstrates that elevated seCCL18 levels identify SSc patients with more severe organ involvement—including pulmonary, cardiac, gastrointestinal and musculoskeletal domains—impaired lung function, and reduced survival. Our study therefore adds to the existing literature by relating seCCL18 levels not only to SSc-ILD and lung function but also to a more comprehensive organ involvement and disease activity (EUSTAR-AI), as well as overall survival in a well-characterised single EUSTAR-centre SSc cohort. Measurement of seCCL18 may contribute to risk stratification and clinical assessment, particularly in patients at risk of progressive pulmonary or cardiac involvement, and may remain informative beyond the early stages of the disease. These findings suggest that seCCL18 may have some clinical utility as a prognostic biomarker associated with widespread disease involvement beyond the lungs, even in long-standing disease. However, the absence of an established cut-off value currently limits its direct clinical implementation, and prospective, large multicentre studies are required to validate its utility and define clinically meaningful thresholds.

## Figures and Tables

**Table 1:** Association between serum CCL18 levels and respiratory involvement in systemic sclerosis.

	**SSc**			**dcSSc**			**lcSSc**		
***n*** **(%)**	**Elevated seCCL18** **(>130 ng/mL)**	**Normal seCCL18** **(≤130 ng/mL)**	***p***	**Elevated seCCL18** **(>130 ng/mL)**	**Normal seCCL18** **(≤130 ng/mL)**	***p***	**Elevated seCCL18** **(>130 ng/mL)**	**Normal seCCL18** **(≤130 ng/mL)**	***p***
SSc-ILD	30/37 * (81.1)	69/114 (60.5)	**0.022**	18/19 * (94.7)	42/64 (65.6)	**0.013**	12/18 (66.7)	27/50 (54)	0.351
FVC < 70%	6/36 * (16.7)	4/114 (3.5)	**0.006**	3/19 (15.8)	4/64 (6.3)	0.189	3/17 * (17.6)	0/50	**0.014**
DLCO < 70%	29/36 * (80.6)	62/114 (54.4)	**0.005**	16/19 * (84.2)	37/64 (57.8)	**0.035**	13/17 (76.5)	25/50 (50)	0.057

seCCL18: serum C-C motif chemokine ligand 18; SSc: systemic sclerosis; dcSSc: diffuse cutaneous systemic sclerosis; lcSSc: limited cutaneous systemic sclerosis; SSc-ILD: systemic sclerosis–associated interstitial lung disease; FVC: forced vital capacity; DLCO: diffusing capacity for carbon monoxide; SD: standard deviation. Data are presented as number of patients; *n* (%). Statistically significant *p*-values (*p* < 0.05) are indicated by asterisks and bold type (χ^2^ or Fisher’s exact test used as appropriate, * *p* < 0.05).

**Table 2:** Clinical manifestations, serological and inflammatory parameters based on the serum CCL18 cut-off levels.

	**SSc**			**dcSSc**			**lcSSc**		
**Clinical Feature,** ***n*** **(%)**	**Elevated** **seCCL18** **(>130 ng/mL)**	**Normal** **seCCL18** **(≤130 ng/mL)**	***p***	**Elevated** **seCCL18** **(>130 ng/mL)**	**Normal** **seCCL18** **(≤130 ng/mL)**	***p***	**Elevated** **seCCL18** **(>130 ng/mL)**	**Normal** **seCCL18** **(≤130 ng/mL)**	***p***
**Musculoskeletal**									
Small joint contracture	24/37 * (64.9)	49/111 (44.1)	**0.029**	14/19 (73.7)	36/62 (58.1)	0.220	10/18 * (55.6)	13/49 (26.5)	**0.027**
TFR	19/37 * (51.4)	31/113 (27.4)	**0.007**	12/19 * (63.2)	23/63 (36.5)	**0.040**	7/18 * (38.9)	8/50 (16)	**0.045**
**Vascular**									
Digital ulcer ever	15/37 (40.5)	40/111 (36)	0.623	7/19 (36.8)	30/62 (48.4)	0.377	8/18 * (44.4)	10/49 (20.4)	**0.049**
**Gastrointestinal**									
GERD	22/37 (59.5)	91/114 * (79.8)	**0.013**	10/19 (52.6)	53/64 * (82.8)	**0.007**	12/18 (66.7)	38/50 (76)	0.442
**Laboratory**									
ATA positive	15/37 * (40.5)	25/114 (21.9)	**0.026**	10/19 (52.6)	22/64 (34.4)	0.151	5/18 * (27.8)	3/50 (6.0)	**0.014**
RNA-Pol III positive	6/37 (16.2)	9/111 (8.1)	0.157	2/19 (10.5)	7/62 (11.3)	0.926	4/18 * (22.2)	2/49 (4.1)	**0.040**
CRP > 5 mg/L	19/37 * (51.4)	22/114 (19.3)	**<0.001**	8/19 * (42.1)	11/64 (17.2)	**0.023**	11/18 * (61.1)	11/50 (22)	**0.002**
ESR > 28 mm/h	14/37 * (37.8)	21/114 (18.4)	**0.015**	8/19 * (42.1)	10/64 (15.6)	**0.014**	6/18 (33.3)	11/50 (22)	0.341
**Other**									
Sicca symptoms	17/37 (45.9)	74/111 * (66.7)	**0.025**	8/19 (42.1)	36/62 (58.1)	0.222	9/18 (50)	38/49 * (77.6)	**0.029**
Currently on immunosuppressants	25/37 * (67.6)	48/114 (42.1)	**0.007**	16/19 * (84.2)	36/64 (56.3)	**0.027**	9/18 * (50)	12/50 (24)	**0.041**

SSc-ILD: systemic sclerosis-associated interstitial lung disease; FVC: forced vital capacity; DLCO: diffusing capacity for carbon monoxide; GERD: gastroesophageal reflux disease; TFR: tendon friction rubs; ATA: anti-topoisomerase I antibody; RNA-Pol III: RNA polymerase III antibody; CRP: C-reactive protein; ESR: erythrocyte sedimentation rate. Data are presented as number of patients; *n* (%). *p*-values were determined by the χ^2^ or Fisher’s exact test used as appropriate. Statistically significant *p*-values (*p* < 0.05) are indicated by asterisk (*) and bold type.

**Table 3:** Univariate analysis and multivariate Cox regression (backward stepwise) analysis of 147 SSc patients.

**Univariate Analysis (Kaplan–Meier)**		
	Log Rank Chi-Square	*p*
Elevated seCCL18 (>130 ng/mL)	6.218	**0.013**
SSc-ILD	7.069	**0.008**
DLCO < 80%	6.047	**0.014**
Arrhythmia (ECG or Holter monitor)	6.659	**0.010**
Six Minute Walk Test below lower limit of normal	14.459	**<0.001**
Small joint contractures (joint < 75% range of motion)	8.061	**0.005**
Subcutaneous calcinosis	4.393	**0.036**
Low BMI (<18.5 kg/m^2^) or weight loss (10% in 1 year) due to malabsorption	4.186	**0.041**
Multivariate Cox regression (backward stepwise)		
	Overall mortality risk HR (95% CI)	*p*
Six Minute Walk Test Reference (per 10% decrease)	1.249 (1.046–1.490)	**0.014**
seCCL18 (per 1-SD increment)	1.789 (1.133–2.824)	**0.013**
Decreasing DLCO (per 10% decrease)	1.942 (1.321–2.857)	**<0.001**

HR: Hazard Ratio; CI: confidence interval; mRSS: modified Rodnan Skin Score; DLCO: diffusing capacity for carbon monoxide in %, CRP: C-reactive protein in mg/L; ESR: erythrocyte sedimentation rate in mm/h; PAH: pulmonary arterial hypertension.

## Data Availability

The datasets generated and/or analyzed during this study are not publicly available due to ethical issues, but are available from the corresponding author on reasonable request.

## Supplementary Materials

The following supporting information can be downloaded at: https://www.mdpi.com/article/10.3390/biom16010136/s1, Figure S1: Serum CCL18 levels of SSc patients sorted by the presence of SSc-ILD; Table S1: STROBE Statement—Checklist of items that should be included in reports of cross-sectional studies; Table S2: Baseline characteristics of patients with systemic sclerosis (SSc); Table S3: Association of serum CCL18 levels with lung function parameters and functional exercise capacity (6-Minute Walk Test) results in SSc; Table S4: Serum CCL18 levels according to organ involvement, autoantibody profile, and inflammatory parameters in SSc.

## Abbreviations

The following abbreviations are used in this manuscript:

ACA	Anticentromere antibody
ACEi	Angiotensin-converting enzyme inhibitor
ACR	American College of Rheumatology
AECG	American–European Consensus Group
ANA	Antinuclear antibody
ANCA	Antineutrophil cytoplasmic antibody
ARB	Angiotensin II receptor blocker
ATA	Anti-topoisomerase I antibody
BMI	Body mass index
CI	Confidence interval
CRP	C-reactive protein
dcSSc	Diffuse cutaneous systemic sclerosis
DLCO	Diffusing capacity for carbon monoxide
DU	Digital ulcer
ECG	Electrocardiography
EF	Ejection fraction
ELISA	Enzyme-linked immunosorbent assay
EMR	Electronic medical record
ESR	Erythrocyte sedimentation rate
EULAR	European League Against Rheumatism
EUSTAR	European Scleroderma Trials and Research Group
EUSTAR-AI	EUSTAR Activity Index
FVC	Forced vital capacity
GERD	Gastroesophageal reflux disease
GI	Gastrointestinal
HC	Healthy control
HR	Hazard ratio
HRCT	High-resolution computed tomography
IQR	Interquartile range
lcSSc	Limited cutaneous systemic sclerosis
LOX	Lysyl oxidase
LVDD	Left ventricular diastolic dysfunction
LVMi	Left ventricular mass index
mPAP	Mean pulmonary arterial pressure
mRSS	Modified Rodnan Skin Score
NHIF	National Health Insurance Fund
PAH	Pulmonary arterial hypertension
PAWP	Pulmonary artery wedge pressure
PVR	Pulmonary vascular resistance
RHC	Right heart catheterization
RNA-Pol III	RNA polymerase III antibody
SD	Standard deviation
seCCL18	Serum C–C motif chemokine ligand 18
SP-D	Surfactant protein D
SSc	Systemic sclerosis
SSc-ILD	Systemic sclerosis–associated interstitial lung disease
TFR	Tendon friction rub
TLC	Total lung capacity
TTE	Transthoracic echocardiography
UCLA-GIT 2.0	University of California Los Angeles Gastrointestinal Tract 2.0 questionnaire
WU	Wood unit
6MWT	Six-minute walk test
