# Supplementary material for: Serum CCL18 May Reflect Multiorgan Involvement with Poor Outcome in Systemic Sclerosis
Source: Biomolecules. 2026 Jan 13;16(1):136. doi: 10.3390/biom16010136 (PMC12838645; doi:10.3390/biom16010136)
Supplement: Supplementary file 1 [file biomolecules-16-00136-s001.zip › biomolecules-4078485-supplementary.pdf]

Table S1. STROBE Statement - Checklist of items that should be included in reports of cross-sectional studies

	Item No	Recommendation	Page No
**Title and abstract**	1	(*a*) Indicate the study's design with a commonly used term in the title or the abstract	1
		(*b*) Provide in the abstract an informative and balanced summary of what was done and what was found	1
**Introduction**			
Background/rationale	2	Explain the scientific background and rationale for the investigation being reported	2
Objectives	3	State specific objectives, including any prespecified hypotheses	2
**Methods**			
Study design	4	Present key elements of study design early in the paper	3-4
Setting	5	Describe the setting, locations, and relevant dates, including periods of recruitment, exposure, follow-up, and data collection	3
Participants	6	(*a*) Give the eligibility criteria, and the sources and methods of selection of participants	3
Variables	7	Clearly define all outcomes, exposures, predictors, potential confounders, and effect modifiers. Give diagnostic criteria, if applicable	3-4
Data sources/ measurement	8*	For each variable of interest, give sources of data and details of methods of assessment (measurement). Describe comparability of assessment methods if there is more than one group	*3-4*
Bias	9	Describe any efforts to address potential sources of bias	3-4, 16
Study size	10	Explain how the study size was arrived at	3
Quantitative variables	11	Explain how quantitative variables were handled in the analyses. If applicable, describe which groupings were chosen and why	3-4
Statistical methods	12	(*a*) Describe all statistical methods, including those used to control for confounding	4
		(*b*) Describe any methods used to examine subgroups and interactions	4
		(*c*) Explain how missing data were addressed	4
		(*d*) If applicable, describe analytical methods taking account of sampling strategy	n.a
		(*e*) Describe any sensitivity analyses	3-4
**Results**			
Participants	13*	(a) Report numbers of individuals at each stage of study—eg numbers potentially eligible, examined for eligibility, confirmed eligible, included in the study, completing follow-up, and analysed	4-14

		(b) Give reasons for non-participation at each stage	4-10
		(c) Consider use of a flow diagram	n.a
Descriptive data	14*	(a) Give characteristics of study participants (eg demographic, clinical, social) and information on exposures and potential confounders	4-10
		(b) Indicate number of participants with missing data for each variable of interest	4-10
Outcome data	15*	Report numbers of outcome events or summary measures	9-10
Main results	16	(*a*) Give unadjusted estimates and, if applicable, confounder-adjusted estimates and their precision (eg, 95% confidence interval). Make clear which confounders were adjusted for and why they were included	4-10
		(*b*) Report category boundaries when continuous variables were categorized	4-10
		(*c*) If relevant, consider translating estimates of relative risk into absolute risk for a meaningful time period	n.a
Other analyses	17	Report other analyses done—eg analyses of subgroups and interactions, and sensitivity analyses	4-10
**Discussion**			
Key results	18	Summarise key results with reference to study objectives	10-12
Limitations	19	Discuss limitations of the study, taking into account sources of potential bias or imprecision. Discuss both direction and magnitude of any potential bias	10-12
Interpretation	20	Give a cautious overall interpretation of results considering objectives, limitations, multiplicity of analyses, results from similar studies, and other relevant evidence	10-12
Generalisability	21	Discuss the generalisability (external validity) of the study results	10-12
**Other information**			
Funding	22	Give the source of funding and the role of the funders for the present study and, if applicable, for the original study on which the present article is based	12

*Give information separately for exposed and unexposed groups.

Table S2. Baseline characteristics of patients with systemic sclerosis (SSc)

Clinical characteristics	SSc n=151	dcSSc n=83	lcSSc n=68	p
Female, n (%)	129 (85.4)	66 (79.5)	63 (92.6)*	**0.023**
Age at enrolment, median years (IQR)	58 (46;64)	54 (40;63)	61 (53.25;65)*	**0.002**
Disease duration at enrolment, median years (IQR)	9 (4;16)	7 (3;13)	12.5 (6.25;17)*	**0.001**
Raynaud's phenomenon, n (%)	146 (96.7)	81 (97.6)	65 (95.6)	0.494
Sclerodactyly, n (%)	146 (96.7)	82 (98.8)	64 (94.1)	0.110
ANA positive, n (%)	114/150 (76.0)	62/82 (75.6)	52 (76.5)	0.902
ACA positive, n (%)	35 (23.2)	10 (12)	25 (36.8)*	**<0.001**
ATA positive, n (%)	40 (26.5)	32 (38.6)*	8 (11.8)	**<0.001**
anti-RNA-Pol III positive, n (%)	15/148 (10.1)	9/81 (11.1)	6/67 (8.8)	0.665
Currently on immunosuppressants, n (%)	73 (48.3)	52 (62.7)*	21 (30.9)	**<0.001**
Arterial hypertension, n (%)	73 (48.3)	31 (37.3)	42 (61.8)*	**0.003**
Currently on ACEi or ARB, n (%)	52 (34.4)	20 (24.1)	32 (47.1)*	**0.003**
Currently on diuretics, n (%)	55 (36.4)	24 (28.9)	31 (45.6)*	**0.034**
**Organ involvement**				
**Respiratory**				
SSc-ILD, n (%)	99 (65.5)	60 (72.3) *	39 (57.4)	**0.008**
FVC<70%, n (%)	10/150 (6.7)	7 (8.4)	3/67 (4.5)	0.334
Oxygen supplementation, n (%)	0	0	0	1.000
**Cardiovascular**				
PAH, n (%)	5 (3.3)	3 (3.6)	2 (2.9)	0.818
Myocardial disease, n (%)	116 (76.8)	58 (69.9)	58 (85.3) *	**0.026**
EF, mean(±SD)	62.5 (±5.1)	62 (±5.6)	63.2 (±4.3)	0.646
EF<50%, n (%)	3/138 (2.2)	3/76 (3.9)	0/62	0.114
LVDD, n (%)	103 (69.7)	50 (60.2)	53 (77.9) *	**0.020**
E/A, median (IQR)	1.0 (0.75;1.29)	1.14 (0.77;1.37) *	0.88 (0.73;1.13)	**0.011**
LVMi, mean(±SD)	101.4 (±21.8)	99.1 (24.4)	104.2 (±17.8)	0.050
Pericardial effusion, n (%)	4/140 (2.9)	3/78 (3.8)	1/62 (1.6)	0.431
**Gastrointestinal**				
Oesophageal involvement, n (%)	51/145 (35.2)	31/81 (38.3)	18/64 (28.1)	0.200
GERD, n (%)	111 (74)	61 (73.5)	50 (73.5)	0.738
Currently on acid-suppressive therapy, n (%)	131 (87.3)	72 (86.7)	59 (86.7)	0.997
Lower GI involvement, n (%)	72 (47.7)	37 (44.6)	35 (51.5)	0.399
UCLA-GIT 2.0 score, median (IQR)	0.21 (0.08;0.46)	0.18 (0.06;0.44)	0.22 (0.08;0.6)	0.646
Weight loss (> 10%) in the past year or BMI <18.5, due to GI involvement, n (%)	9 (6.0)	5 (6.0)	4 (5.9)	0.959
**Vascular**				
Digital ulcers ever, n (%)	56 (37.1)	38 (45.8) *	18 (26.5)	**0.015**
Telangiectasia, n (%)	92 (60.9)	47 (56.6)	45 (66.2)	0.231
Amputation ever, n (%)	9 (6.0)	7 (8.4)	2 (2.9)	0.253
**Musculoskeletal and skin**				
mRSS, median (IQR)	7 (4;12)	10 (6;14)*	4 (3;6)	**<0.001**
mRSS>14, n (%)	26 (17.2)	26 (31.3)	0	**<0.001**
Arthritis current, n (%)	15 (10)	9 (10.8)	6 (8.8)	0.662
Small joint contracture, n (%)	83 (55.3)	55 (66.3) *	28 (41.2)	**0.002**
Large joint contracture, n (%)	24 (16)	14 (16.9)	10 (14.5)	0.718
Sicca symptoms, n (%)	92 (60.9)	45 (54.2)	47 (69.1)	0.062
Proximal muscle weakness, n (%)	21 (16.5)	12 (14.5)	9 (13.2)	0.829

Calcinosis, n (%)	30/148 (20.3)	14/81 (17.3)	16/67 (23.9)	0.320
Tendon friction rub, n (%)	50/150 (33.3)	35/82 (42.7) *	15 (22.1)	**0.008**
**Renal**				
Scleroderma renal crisis, n (%)	1 (0.7)	0	1 (1.5)	0.270
Renal replacement therapy, n (%)	0	0	0	1.000

SSc: systemic sclerosis; dcSSc: diffuse cutaneous systemic sclerosis; lcSSc: limited cutaneous systemic sclerosis; SSc-ILD: systemic sclerosis–associated interstitial lung disease; FVC: forced vital capacity; DLCO: diffusing capacity for carbon monoxide; PAH: pulmonary arterial hypertension; EF: ejection fraction; LVDD: left ventricular diastolic dysfunction; E/A: early-to-late diastolic filling ratio; LVMi: left ventricular mass index; GERD: gastroesophageal reflux disease; GI: gastrointestinal; UCLA-GIT 2.0: University of California Los Angeles Gastrointestinal Tract 2.0 questionnaire; BMI: body mass index; mRSS: modified Rodnan Skin Score; ANA: antinuclear antibody; ACA: anticentromere antibody; ATA: anti-topoisomerase I antibody; RNA-Pol III: RNA polymerase III antibody; ACEi: Angiotensin-converting enzyme (ACE) inhibitors; ARB: Angiotensin II receptor blocker; CRP: C-reactive protein; ESR: erythrocyte sedimentation rate; TFR: tendon friction rub; SD: standard deviation; IQR: interquartile range. Data are presented as n (%), median (IQR), or mean ± SD, as indicated. Statistical test: χ^2 test, Fisher's Exact test and Mann-Whitney U test as appropriate, level of significance: $p<0.05$. Significant differences between dcSSc and lcSSc are marked with asterisks and bold characters.

Table S3. Association of serum CCL18 levels with lung function parameters and functional exercise capacity (6-Minute Walk Test) results in SSc

	**Elevated seCCL18 (>130 ng/ml)**	**Normal seCCL18 (≤130 ng/ml)**	**p**
**Total cohort (n=151)**			
	n=37	n=114	
FVC (%),mean ± SD	**94±21***	104±19	**0.006**
DLCO (%),mean ± SD	**59±16***	66±18	**0.015**
TLC (%),mean ± SD	**104±19***	115±19	**0.002**
6MWT distance, meters (mean ± SD)	444.6±140.6	433.6±133.0	0.570
6MWT predicted value (%) (mean ± SD)	84.7±23.8	79.6±21.9	0.205
Reduced 6MWT performance (<LLN), n (%)	10 (27.0)	40 (35.1)	0.365
**dcSSc (n=83)**			
	n=19	n=64	
FVC (%),mean ± SD	94±21	101±19	0.188
DLCO (%),mean ± SD	57±13	66±18	0.067
TLC (%), mean ± SD	**98±15***	111±19	**0.004**
6MWT distance, meters (mean ± SD)	491.2±126.5	445.9±121.4	0.072
6MWT predicted value (%) (mean ± SD)	**93.7±22.7***	77.5±18.6	**0.003**
Reduced 6MWT performance (<LLN), n (%)	3 (15.8)	**27 (42.2)***	**0.035**
**lcSSc (n=68)**			
	n=18	n=50	
FVC (%), mean ± SD	**95±21***	109±19	**0.007**
DLCO (%), mean ± SD	61±19	67±17	0.102
TLC (%), mean ± SD	111±21	120±17	0.100
6MWT distance, meters (mean ± SD)	398±141.8	418.0±146.3	0.536
6MWT predicted value (%) (mean ± SD)	75.6±21.9	82.3±25.4	0.243
Reduced 6MWT performance (<LLN), n (%)	7 (38.9)	13 (26.0)	0.303

FVC: forced vital capacity; DLCO: diffusing capacity for carbon monoxide; TLC: total lung capacity; 6MWT: 6-Minute Walk Test; seCCL18: serum C-C motif chemokine ligand 18; SSc: systemic sclerosis; dcSSc: diffuse cutaneous systemic sclerosis; lcSSc: limited cutaneous systemic sclerosis. Data are presented as n (%) or mean ± SD, as indicated. Statistical test: χ^2 test, Fisher's Exact test and Mann-Whitney U test as appropriate, level of significance: p<0.05.

Supplementary Results: seCCL18 and 6MWT performance

Investigating the functional exercise capacity by 6MWT, we observed that normal seCCL18 levels in dcSSc patients were associated with a lower percentage of the predicted value (%) of 6MWT distance (77±19 % vs. 94±23 %, p=0.003). In dcSSc, reduced 6MWT performance (<LLN) was more frequent among patients with normal seCCL18 (27/64, 42% vs. 3/19, 16%, χ^2 test, p=0.035). No similar associations were observed in either the total SSc cohort or the lcSSc subset (Table S1).

Supplementary Results: seCCL18 levels in SSc according to SSc-ILD Status

Patients with SSc-ILD had significantly higher seCCL18 levels than those without ILD in the total cohort (p<0.001) (Figure S1a). This difference remained significant within the dcSSc subset as well (p<0.001) (Figure S1b). In dcSSc, patients with ILD showed significantly higher seCCL18 levels compared with those without ILD (Figure S1b). Although the difference between lcSSc patients with and without ILD did not reach statistical significance, lcSSc-ILD patients had higher seCCL18 concentrations than dcSSc patients without ILD (p<0.005, Figure S1b).

Figure S1a, S1b. Serum CCL18 levels of SSc patients sorted by the presence of SSc-ILD

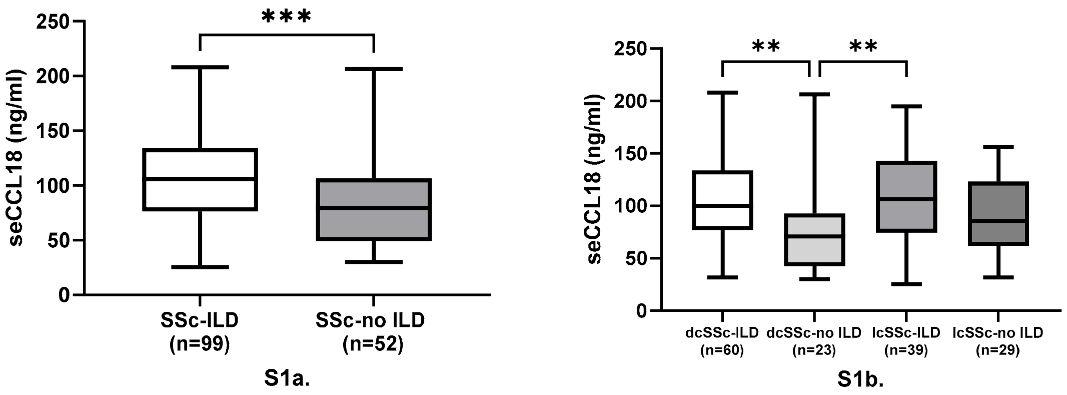

(S1a) Serum CCL18 (seCCL18) concentrations in patients with systemic sclerosis with and without interstitial lung disease (SSc-ILD vs. SSc-no ILD). (S1b) Serum CCL18 concentrations in diffuse and limited cutaneous systemic sclerosis (dcSSc, lcSSc) patients with and without ILD. Data are shown as box-and-whisker plots indicating the median, interquartile range, and minimum–maximum values. p-values were calculated using the Mann–Whitney U test and Kruskal-Wallis test as appropriate. Asterisks represent statistical significance levels: p < 0.005 (**), p < 0.001 (***).

seCCL18: serum CC chemokine ligand 18; SSc: systemic sclerosis; ILD: interstitial lung disease; dcSSc: diffuse cutaneous systemic sclerosis.

Table S4. Serum CCL18 levels according to organ involvement, autoantibody profile, and inflammatory parameters in SSc

	SSc			dcSSc			lcSSc		
**Clinical feature present**	**Yes (seCCL18 mean±SD)**	**No (seCCL18 mean±SD)**	**p**	**Yes (seCCL18 mean±SD)**	**No (seCCL18 mean±SD)**	**p**	**Yes (seCCL18 mean±SD)**	**No (seCCL18 mean±SD)**	**p**
**Respiratory**									
SSc-ILD	108.6 ±42.9* (n=99)	83.4 ±39.0 (n=52)	**<0.001**	109.2 ±43.2* (n=60)	73.7 ±40.8 (n=23)	**<0.001**	107.7 ±42.9 (n=39)	91.2 ±36.4 (n=29)	0.115
FVC < 70%	132.6 ±46.5* (n=10)	97.3 ±42.1 (n=140)	**0.023**	117.9 ±45.6 (n=7)	97.7 ±45.1 (n=76)	0.207	166.8 ±31.6* (n=3)	96.9 ±38.6 (n=64)	**0.015**
DLCO < 70%	109.1 ±44.7* (n=91)	85.2 ±36.5 (n=59)	**<0.001**	109.9 ±45.8* (n=53)	80.7 ±38.3 (n=30)	**0.002**	107.9 ±43.9 (n=38)	89.8 ±34.6 (n=29)	0.092
**Cardiac**									
Myocardial disease	104.8 ±41.8* (n=116)	83.8 ±44.2 (n=35)	**0.008**	107.0 ±43.7* (n=58)	81.7 ±44.6 (n=25)	**0.011**	102.6 ±40.1 (n=58)	89.1 ±45.1 (n=10)	0.387
LVDD	107.1 ±40.5* (n=103)	84.5 ±45.0 (n=48)	**<0.001**	110.0 ±41.2* (n=50)	83.2 ±46.9 (n=33)	**0.003**	104.5 ±40.1 (n=53)	87.2 ±42.0 (n=15)	0.171
**GI**									
Oesophageal involvement (dysmotility, stricture)	110.7 ±38.3* (n=49)	93.3 ±43.1 (n=96)	**0.009**	107.7 ±37.9* (n=31)	91.1 ±46.3 (n=50)	**0.039**	115.8 ±39.5 (n=18)	95.8 ±39.8 (n=46)	0.057
GERD	96.3 ±39.6 (n=113)	110.8 ±51.3 (n=38)	0.154	92.2 ±40.0 (n=63)	122.0 ±53.9* (n=20)	**0.034**	101.4 ±39.0 (n=50)	98.4 ±46.7 (n=18)	0.718
**Laboratory**									
ATA	114.0 ±41.8* (n=40)	94.9 ±42.7 (n=111)	**0.015**	109.4 ±42.8 (n=32)	93.1 ±46.0 (n=51)	0.089	132.5 ±33.8* (n=8)	96.4 ±40.0 (n=60)	**0.019**
ACA	93.55 ±39.8 (n=35)	101.9 ±44.1 (n=116)	0.360	76.4 ±51.8 (n=10)	102.5 ±43.7* (n=73)	**0.037**	100.4 ±32.5 (n=25)	100.8 ±45.3 (n=43)	0.919
RNA-Pol III	114.3 ±53.1 (n=15)	98.2 ±42.3 (n=133)	0.240	93.1 ±56.7 (n=9)	100.2 ±44.7 (n=72)	0.471	146.2 ±26.5* (n=6)	95.8 ±39.5 (n=61)	**0.004**
CRP >5 mg/l	127.9 ±45.3* (n=41)	89.5 ±37.5 (n=110)	**<0.001**	129.7 ±50.7* (n=19)	90.4 ±39.6 (n=64)	**0.004**	126.4 ±41.3* (n=22)	88.3 ±34.7 (n=46)	**<0.001**
ESR>28 mm/h	126.0 ±40.5* (n=35)	92.1 ±40.9 (n=116)	**<0.001**	130.3 ±47.0* (n=18)	90.8 ±41.1 (n=65)	**0.002**	121.4 ±33.1* (n=17	93.7 ±41.1 (n=51)	**0.013**

seCCL18: serum CC chemokine ligand 18; SSc: systemic sclerosis; dcSSc: diffuse cutaneous systemic sclerosis; lcSSc: limited cutaneous systemic sclerosis; LVDD: left ventricular diastolic dysfunction; GI: gastrointestinal; GERD: gastroesophageal reflux disease; ATA: anti-topoisomerase I antibody; ACA: anticentromere antibody; RNA-Pol III: RNA polymerase III antibody; CRP: C-reactive protein; ESR: erythrocyte sedimentation rate; SD: standard deviation. The seCCL18 levels are presented as mean ± SD. Statistically The p-values were calculated using the Mann–Whitney U test. Statistically significant p-values ($p < 0.05$) are indicated by asterisks and bold type.
